# The effect of gravity-induced preload change on the venous excess ultrasound (VExUS) score and internal jugular vein Doppler in healthy volunteers

**DOI:** 10.1186/s40635-023-00504-8

**Published:** 2023-04-14

**Authors:** Jon-Emile S. Kenny, Ross Prager, Philippe Rola, Garett McCulloch, Joseph K. Eibl, Korbin Haycock

**Affiliations:** 1 Health Sciences North Research Institute, 56 Walford Rd, Sudbury, ON P3E 2H2 Canada; 2 Flosonics Medical, Toronto, ON Canada; 3 Division of Critical Care Medicine, Western University, London, ON Canada; 4 Intensive Care Unit, Santa Cabrini Hospital, Montreal, QC Canada; 5 Northern Ontario School of Medicine, Sudbury, ON Canada; 6 Department of Emergency Medicine, Riverside University Health System Medical Center, Moreno Valley, CA USA

**Keywords:** Venous excess ultrasound score, Venous congestion, Fluid tolerance, Internal jugular vein, Healthy volunteer

## Abstract

**Background:**

The venous excess ultrasound (VExUS) score is a multi-organ Doppler approach to assess venous congestion. Despite growing use of VExUS in research and clinical practice, other veins can be visualized to assess for venous hypertension, which may overcome acquisition barriers of the VExUS exam. In this pilot, observational study, we used a wearable Doppler ultrasound to assess the relationship between jugular venous Doppler and the VExUS score under different preload conditions. We hypothesized that jugular Doppler morphology would accurately distinguish preload conditions, that it would most closely relate to the hepatic venous Doppler morphology in the fully supine position and that the VExUS score would be influenced by preload condition.

**Results:**

We recruited 15 healthy volunteers with no cardiovascular history. Preload change was achieved using a tilt-table with three positions: supine, fully upright, and 30-degree head-down tilt. In each position, a VExUS score was performed; furthermore, inferior vena collapsibility and sphericity index were calculated. At the same time, jugular venous Doppler was captured by a novel, wireless, wearable ultrasound system. A continuous jugular venous Doppler morphology was 96% accurate for detecting the low preload condition. The jugular venous Doppler morphology was highly correlated with the hepatic vein, but only in the supine position. Gravitational position did not significantly affect the sphericity index or the VExUS score.

**Conclusions:**

The jugular vein Doppler morphology was able to accurately distinguish low from high preload conditions in healthy volunteers. Comparisons between VExUS Doppler morphologies and other veins should occur in the supine position when gravitational pressure gradients are minimized; finally, different preload conditions in healthy subjects did not affect the VExUS score.

## Background

A criticism of the ‘liberal’ intravenous (IV) fluid resuscitative approach is that excessive IV volume risks raising venous pressure, which may impair organ perfusion [1–3]. Accordingly, associations between positive fluid balance and poor outcome may be partly explained by ‘congestive’ pathophysiology [4]. Historically, intensivists have monitored venous hypertension invasively by measuring the central venous pressure [5]. However, there is a trend in the twenty-first century to monitor hemodynamics non-invasively, especially via ultrasound [6]. With increasing awareness of venous Doppler ultrasound and its relationship to venous congestion, the venous excess ultrasound (VExUS) was developed [3].

The VExUS score has been associated with adverse outcomes in a number of critically ill populations, particularly with respect to acute kidney injury [7–9]. Though the VExUS score focuses on the spectral Doppler morphology of three intra-abdominal veins (i.e., hepatic, portal and intra-renal), venous spectral Doppler of the superior and inferior vena cavae [10, 11], femoral vein [12] and internal jugular vein [13–15] have all previously been linked to right heart function. Regarding the jugular vein, we have described rapid spectral Doppler changes with preload [6, 16, 17]—observations facilitated by a wireless, wearable Doppler ultrasound system that continuously displays both the common carotid arterial and internal jugular venous Doppler spectra [16–20]. Given the simplicity of acquiring jugular waveforms using a wearable Doppler ultrasound system, we wondered how jugular Doppler would compare to the VExUS score during preload changes in healthy subjects.

In this pilot investigation, we evaluated three basic hypotheses. For our primary outcome, we expected that the internal jugular vein Doppler morphology would be highly accurate for detecting gravity-induced changes in preload. For our secondary outcome, we predicted that the Doppler morphologies of the internal jugular and hepatic veins would correlate most in the supine position given their anatomical proximities to the right atrium and the absence of a gravitational pressure gradient between them when lying flat. For our tertiary outcome, we anticipated that gravity-induced changes in the right atrial pressure would move the jugular venous Doppler and the VExUS score in physiologically opposing directions given that the jugular vein is anatomically above the right atrium while the 3 veins in the VExUS score are anatomically below the right atrium.

## Methods

### Clinical setting

We recruited healthy, adult, volunteers who were clinically euvolemic. We used a convenience sample in a local physiology lab, a power calculation was not performed as this was a pilot study. Written and informed consent was obtained for all subjects and the study was reviewed and approved by the Research Ethics Board of Health Sciences North (IRB number and date of approval: CR00351324, Mar. 21, 2022). The procedures followed were in accord with the local ethical standards of the committee on human experimentation and with the Helsinki Declaration of 1975. Exclusion criteria were known cardiovascular history and/or taking regular cardiovascular medications.

### Venous excess ultrasound score

The VExUS score has been previously described [3]; it comprises measurements of the end-expiratory inferior vena cava (IVC) dimension in addition to Doppler signals from the hepatic, portal and intra-renal veins. A normal score consists of an IVC diameter less than 2 cm, a systolic greater than diastolic wave in the hepatic vein Doppler, a continuous portal vein Doppler (i.e., with pulsatility less than 30%) and a continuous intra-renal vein pattern.

### Internal jugular vein Doppler

Internal jugular vein Doppler was obtained using a novel, wireless, wearable Doppler ultrasound system. As previously described [18, 21, 22], the common carotid artery and internal jugular vein Doppler signals are obtained by simultaneously-acquired visual and audio cues from the wearable system. Once common carotid and internal jugular venous Doppler signals were seen and heard, the wearable Doppler was adhered in place on the subject’s neck; jugular vein Doppler was monitored throughout the protocol. Doppler morphology was judged at end-expiration and synchronous with the hepatic vein recordings.

**Fig. 1:**
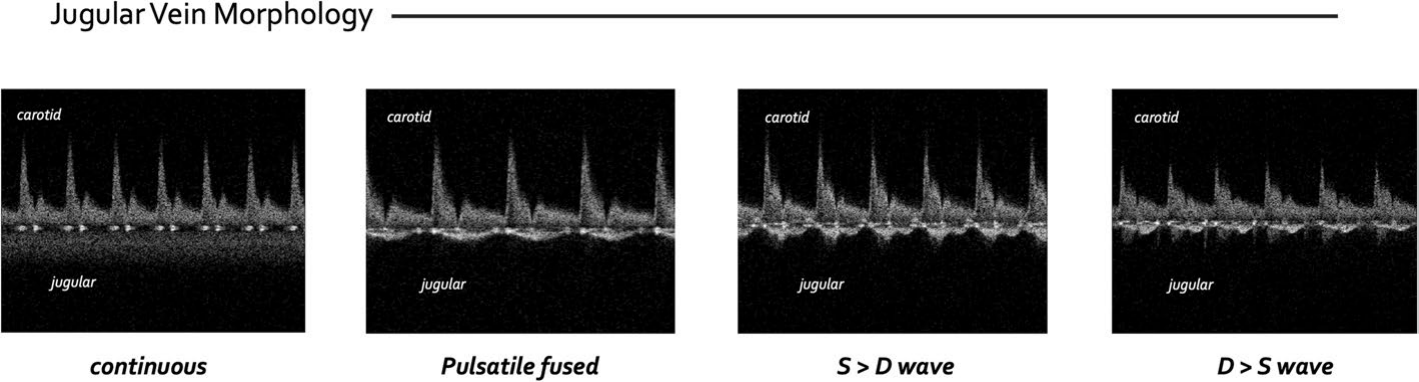
Internal jugular vein scoring system. 0 = continuous; 1 = pulsatile fused or S wave > D wave; 2 = D > S wave

Internal jugular vein Doppler morphology was stored for blind, independent assessment by R.P., P.R., and K.H. The jugular morphology was scored based upon its qualitative pattern, a modification of that described by Iida et al. and Tang for the intra-renal vein [23, 24]. The jugular Doppler scoring was as follows: 0 = continuous; 1 = pulsatile fused or S wave > D wave or; 2 = D wave > S wave (Fig. 1). Any disagreement was resolved by consensus between the 3 experts in venous Doppler.

### Tilt table protocol

Each protocol consisted of three gravitational positions: supine, fully upright and head-down tilt, 30 degrees below the horizon. At each position, a full VExUS score was obtained by one expert in the field (R.P., P.R., K.H.) and completed within 3–5 min of each preload change. Each protocol began in the supine position with baseline measurements obtained; these included the VExUS score, the inferior vena cava sphericity index and continuous internal jugular venous Doppler from the wearable ultrasound. After supine measurements were complete, the second gravitational position for all subjects was the fully upright position; all ultrasound measures were then repeated and the subject returned to the supine position for at least a 3 min ‘wash-out’ period. Thereafter, the subject was tilted to 30 degrees head-down and all ultrasound measures repeated. Thus, the participant had VExUS scores, sphericity indices (i.e., the ratio of the short-to-long axes of the IVC in cross section) [25] and internal jugular vein Doppler measurements repeated in each tilt-table position.

### Analysis of measurements

The analysis was completed in three basic parts. First, preload was dichotomized into low (i.e., fully upright) and high (i.e., head-down tilt) preload conditions; the blinded jugular vein evaluations were matched to these conditions, and the accuracy of the continuous jugular pattern for detecting low preload was calculated. To do this, we defined the following matrix:

(A) true positive occurred when there was low preload and continuous jugular Doppler; (B) true negative occurred when there was high preload with a pulsatile jugular Doppler; (C) false negative occurred when there was low preload with a pulsatile jugular morphology and (D) false positive occurred when there was high preload with continuous Doppler morphology. Overall accuracy was calculated as the true positives plus true negatives divided by the total number of observations. Second, the internal jugular vein Doppler morphology was compared to the hepatic vein within each individual at each gravitational position (i.e., supine, upright and head down). Finally, the total VExUS score for each subject in each gravitational position was also calculated.

### Statistical analysis

A Fleiss Kappa was calculated to assess agreement of jugular vein Doppler morphology between the blinded observers. The effect of position on end-expiratory IVC diameter, sphericity index and portal vein pulsatility index were all compared using a Wilcoxon signed-rank test with significance defined as *p* < 0.05.

## Results

15 adult volunteers were studied; 8 were women. One male subject was entirely excluded because of lost ultrasound image uploads; therefore, the analysis comprises 14 total adults. The baseline characteristics of the healthy volunteers included in the final analysis are listed in Table 1.

**Table 1:** Baseline characteristics and measures in the supine position

*n* = 14	Mean	std
Patient Age	29.6	± 9.3
Patient Height (m)	1.7	± 0.1
Patient Weight (kg)	69.1	± 16.0
BMI (kg/m^2^)	23.1	± 4.2
MAP (mmHg)	96.3	± 10.6
HR (bpm)	73.5	± 10.6
Systolic Blood Pressure (mmHg)	127.7	± 16.3
ccFT (ms)	314.0	± 25.2

M is meters, kg/m^2^ is kilograms per meters-squared, mmHg is millimeters of mercury, bpm is beats per minute, ccFT is corrected flow time of the carotid artery, ms is milliseconds

### Diagnostic accuracy of jugular vein Doppler ultrasound for detecting low preload

**Fig. 2:**
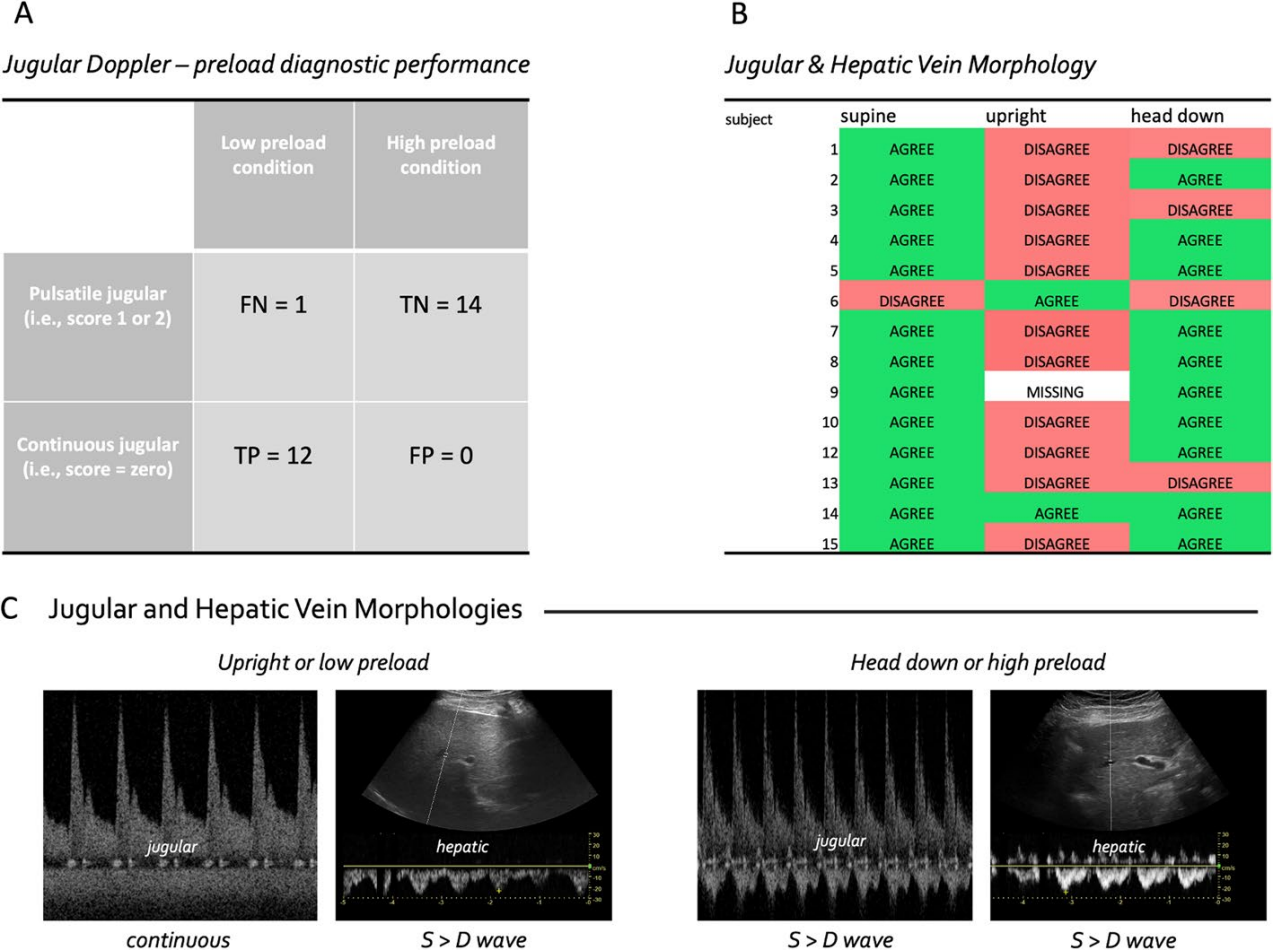
Effect of gravitational preload change on jugular and hepatic veins. **A** 2 × 2 diagnostic accuracy matrix for jugular morphology versus preload, FN is false negative, TN is true negative, TP is true positive and FP is false positive **B** agreement between the jugular vein and hepatic vein morphologies at different preload conditions. **C** Examples of jugular and hepatic vein Doppler at low (left most) versus high (right most) preload conditions

Based on the matrix defined above, there were 12 true positives, 14 true negatives and 27 total observations. Therefore, the total accuracy was 96% (Fig. 2A). There was a single false negative which occurred when a single subject had a pulsatile jugular vein in the upright position. The Fleiss Kappa inter-observer coefficient was very good (0.83) for the three blinded experts.

### Effect of gravitational position on jugular and hepatic vein Doppler

**Fig. 3:**
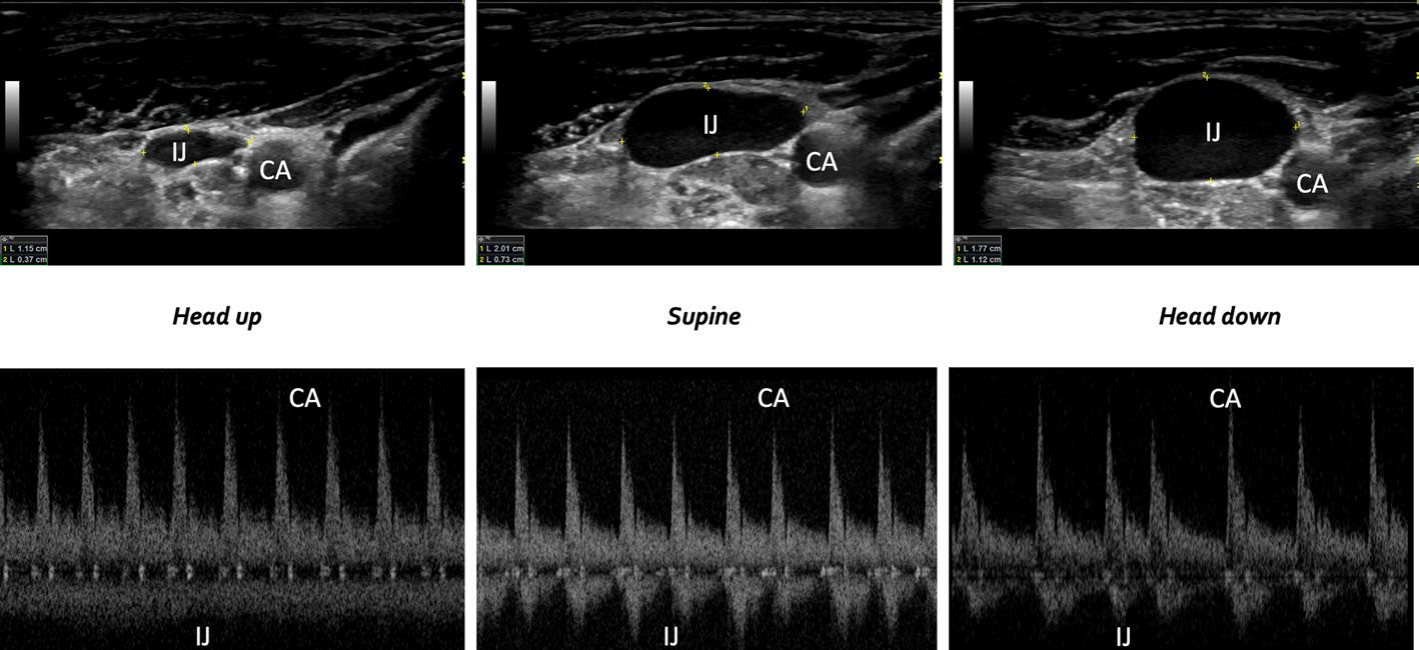
The relationship between B-mode images and Doppler spectra at three preload conditions in a single subject. CA is carotid artery and IJ is internal jugular

**Fig. 4:**
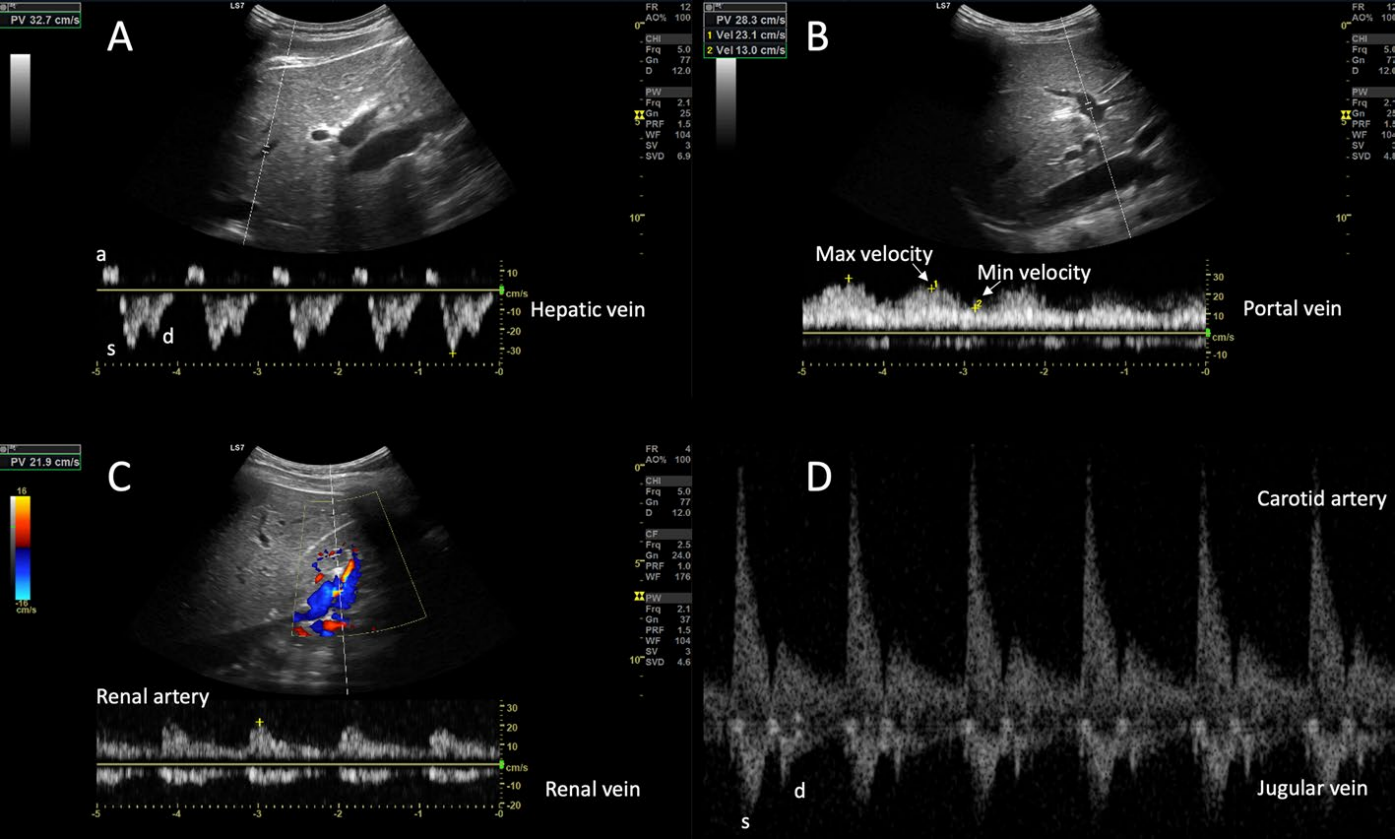
Example of venous scoring during high preload condition. **A** The hepatic vein Doppler at the bottom with labeled a, s and d waves showing an S > D morphology. **B** The portal vein showing the maximum and minimum velocities use to calculate the portal pulsatility index. **C** The renal artery and vein revealing a ‘pulsatile fused’ or ‘short end-diastolic pause’ morphology. **D** The carotid artery and jugular venous spectra from the wearable Doppler. The jugular has an S > D morphology most comparable to the hepatic vein

The jugular vein Doppler morphology was scored as ‘continuous’ in 92% of subjects in the fully upright position, whereas this fraction fell to 7% and 0% in the supine and head-down positions, respectively. By contrast, the hepatic vein Doppler morphology was scored as ‘continuous’ in 21% of subjects in the fully upright position, as well as 21% in and 29% in the supine and head-down positions, respectively. Per the scoring classification scheme described above, the jugular and hepatic vein morphologies agreed 93%, 15% and 71% in the supine, fully upright and head-down positions, respectively (Fig. 2B, C). In one subject, we performed B-mode imaging of the internal jugular vein relative to the common carotid artery at all three gravitational positions (Fig. 3). Figure 4 shows the hepatic, portal, renal and jugular veins during the high preload (i.e., head-down) condition in one subject.

### Effect of gravitational position on VExUS and its components

A full VExUS score was obtained in all subjects. In all but two subjects, the end-expiratory IVC diameter remained below 2 cm in all gravitational positions; thus, the VExUS score remained zero throughout for 86% of subjects. In one of the two subjects in whom IVC was above 2 cm, it remained so across all gravitational positions; this subject had normal hepatic and intra-renal vein Doppler morphologies across all positions, though the subject’s portal vein did become more pulsatile during head-down position, generating a VExUS = 1. In the second subject, IVC was greater than 2 cm only the in upright and head down position with otherwise normal venous Doppler throughout.

**Fig. 5:**
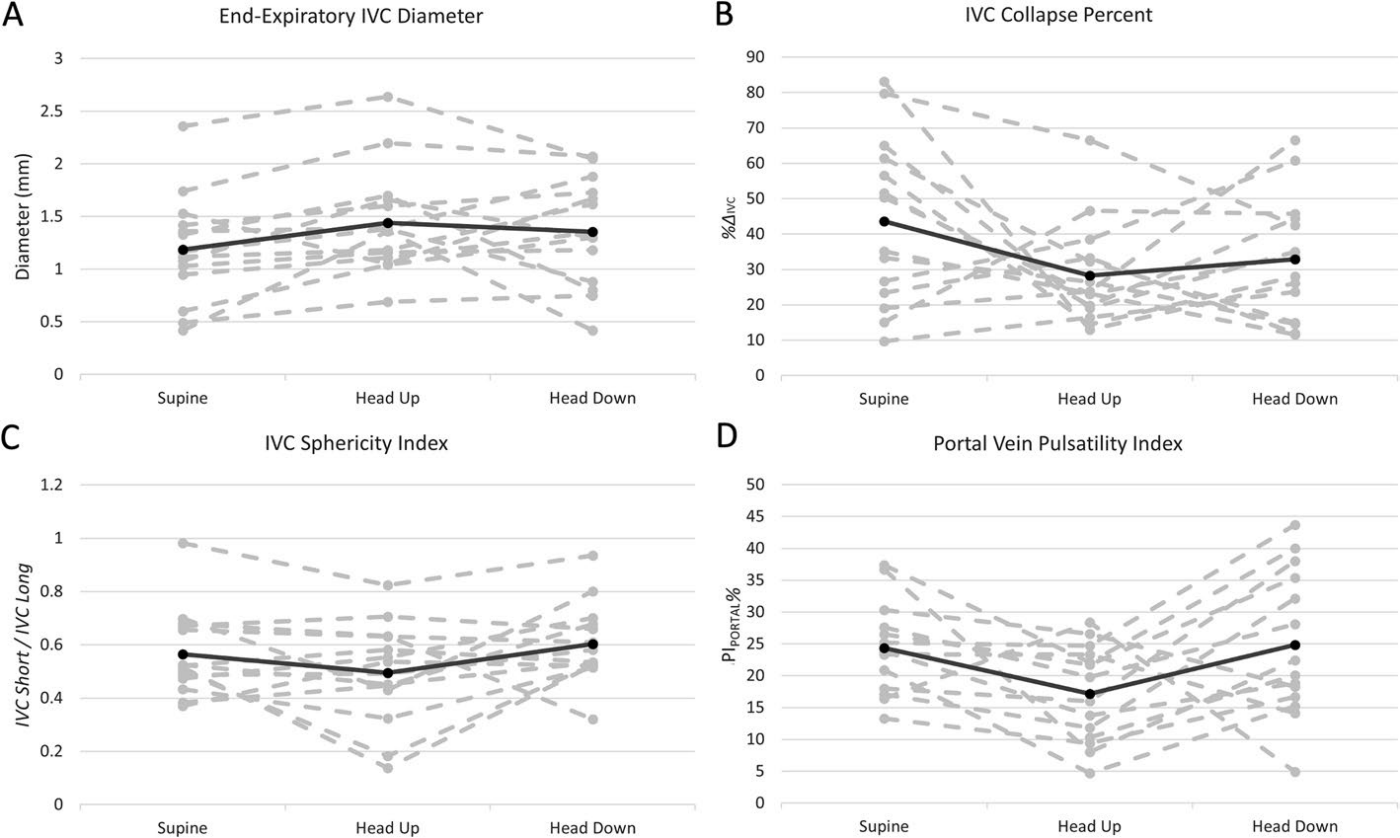
The effect of preload on the inferior vena cava and portal pulsatility. Each dashed grey line indicates a single subject, the black line is the average of all subjects. **A** End-expiratory inferior vena cava (IVC) diameter; **B** IVC collapse; **C** IVC sphericity index and **D** the portal vein pulsatility index

There was no statistically significant change in the sphericity index, or IVC collapsibility index across gravitational changes. There was a statistically significant increase in the end-expiratory IVC diameter from supine to upright (Table 2 and Fig. 5).

**Table 2:** Inferior vena cava and portal vein measures

	Supine				Head up				Head down			
	IVC_EE_	IVC_%_	SI_IVC_	PI_PORTAL_	IVC_EE_	IVC_%_	SI_IVC_	PI_PORTAL_	IVC_EE_	IVC_%_	SI_IVC_	PI_PORTAL_
Mean	1.18	43.57	0.56	24.35	1.44	28.3	0.49	17.18	1.36	32.83	0.60	24.84
Stdev	0.51	23.78	0.16	7.45	0.50	14.54	0.19	18.45	0.51	17.54	0.15	11.86

Supine versus head-up				Supine versus head-down				Head-up versus head-down			
IVC_EE_	IVC_%_	SI_IVC_	PI_PORTAL_	IVC_EE_	IVC_%_	SI_IVC_	PI_PORTAL_	IVC_EE_	IVC_%_	SI_IVC_	PI_PORTAL_
+ 0.25*	− 15.27	− 0.07	− 7.16	+ 0.17	− 10.74	+ 0.04	+ 0.49	− 0.08	+ 4.53	+ 0.11	+ 7.66

*p* < 0.01^*^

Venous measures and their change with preload. All values represent absolute change. IVC_EE_ is end-expiratory IVC diameter in centimeters, IVC_%_ is the inspiratory collapse of the IVC, SI_IVC_ is the sphericity index of the IVC and PI_PORTAL_ is the pulsatility index of the portal vein in %

## Discussion

In this pilot, physiology study assessing the impact of gravitationally modified preload conditions on venous Doppler patterns in healthy volunteers, we noted several clinically relevant findings. First, we found that jugular vein Doppler morphology had high diagnostic accuracy to detect gravity-induced preload condition. Second, the Doppler morphologies of the internal jugular and hepatic veins were most closely related in the supine position. Finally, and contrary to expectation, positional change had very little effect on the overall VExUS score, though end-expiratory IVC diameter increased very slightly in the upright position.

The Doppler morphology of the jugular vein had high diagnostic accuracy to dichotomize pre-load into “high” and “low” categories, which could aid in the rapid phenotyping of shock for acutely decompensating patients. A clinical extrapolation of our ‘true positive’ definition is a patient with low preload (e.g., normal, upright physiology, volume loss, septic venodilation) who is also observed to have continuous jugular Doppler morphology. On the other hand, a ‘true negative’ is a patient with high preload (e.g., volume expansion, cardiac dysfunction) who is observed to have at least an S > D wave jugular morphology. Furthermore, jugular Doppler could help clinicians assess the effect of preload during functional hemodynamic monitoring, as we have previously reported [6, 16, 17, 19]. These observations are facilitated by a wireless, wearable Doppler ultrasound system that continuously insonates the internal jugular vein. That over 90% of healthy volunteers displayed a continuous waveform, which fell to 7% when supine and 0% when head-down, suggested that jugular vein pulsatility qualitatively estimates preload. Notably, none of the intra-abdominal venous Doppler signals correlated with gravity-mediated preload change. For instance, the hepatic vein remained pulsatile in roughly three-quarters of all subjects in each position. This fits previous data that suggest hepatic vein Doppler does not correlate well with right atrial pressure [26]. We cannot answer the mechanism for these observations, though we note that the degree to which a vein’s Doppler signal assumes the right atrial pressure trace is mediated by its volume, compliance, transmural pressure, as well as physical and gravitational relation to the right heart. Given that the surrounding pressure of the jugular vein is atmosphere (rather than intra-hepatic or intra-renal), we suspect that jugular transmural pressure is closest to what would be measured by a central venous catheter zeroed to atmosphere [27]. The continuous nature of the jugular signal in the upright position likely reflects a collapsed or ellipsoid vein at the level of the wearable transducer (Fig. 3). Then, as the central venous pressure rises and the vein rounds out, a pulsatile waveform is observed. Fundamentally, the jugular venous Doppler morphology is a sonographic transduction of the venerable jugular venous pressure (JVP) examination.

Second, as anticipated, the morphology of the internal jugular vein was closely related to the hepatic vein in healthy volunteers, but only when supine. When lying flat, there was only a single disagreement between the jugular and hepatic vein morphologies. In this particular instance, the hepatic Doppler was continuous while the jugular had features consistent with inspiratory collapse, but with pulsatile qualities during expiration. Overall, the greatest disagreement between the hepatic and jugular veins was in the upright position, when the jugular vein was almost always continuous and the hepatic vein pulsatile. This makes sense given the anatomical relationships between the right atrium and the jugular and hepatic veins when upright. Clinically, these observations are important for further venous Doppler research; that is, relating venous morphologies is best achieved in the supine position when gravitational pressure gradients are minimized.

Finally, and somewhat unexpectedly, the overall VExUS score was resistant to gravitational preload changes in healthy volunteers. The primary reason for this was that the vast majority of subjects maintained an IVC diameter below 2 cm in all positions. Nevertheless, the Doppler vein morphologies changed very little individually as well. The hepatic vein Doppler was pulsatile in approximately 75% of subjects in each of the upright, supine and head-down positions. Neither the intra-renal nor hepatic veins showed any clinically significant qualitative changes with gravitational preload variation; in line with this, IVC collapse, portal vein pulsatility and the IVC sphericity indices were not significantly different between positions. While we cannot be sure that these results would be replicated in patients with pathological changes in volume status, intra-abdominal pressure or cardiac function [28], the clinical implication is that the overall VExUS score is resistant to preload change induced by gravity in clinically euvolemic healthy subjects with intact cardiovascular reflexes (i.e., not anesthetized or on cardiovascular medications). Because our observations cannot be generalized to critically-ill patients, gravitational position should still be considered when interpreting the VExUS score, especially in light of the work of Hermansen and colleagues as described below [28]. Nevertheless, our data remain relevant for intensivists because understanding normal, expected, physiology is the foundation for interpreting pathophysiology. As described in a recent, narrative review, the absence of significant change in the VExUS score could have predictive value in the critically-ill, for example, by delineating specific hemodynamic phenotypes defined by a “Doppler Starling curve” [6]. Our data in healthy volunteers support this framework [6]. More specifically, a PLR or Trendelenburg position coupled with the *absence of change* in the VExUS score (and/or a jugular Doppler pattern increasing beyond an S > D wave) could represent a good hemodynamic phenotype (e.g., low risk of weaning-induced cardiogenic edema), especially in conjunction with measures of rising stroke volume [6]. Additional study in the critically-ill population is planned.

Our study has several important limitations. We did not actually measure the central venous pressure or objectively qualify preload. Nevertheless, gravitational changes are well-accepted methods for increasing right heart filling; indeed, this is the rationale for both passive leg raising [29] and the Trendelenburg position [30–32]. Furthermore, in one subject, we performed simultaneous B-mode imaging of the internal jugular vein and common carotid artery in all three positions (Fig. 3). As expected, the area of the jugular vein increased relative to the carotid from upright to supine to head down; the ratio of the jugular-to-carotid area is known to correlate with central venous pressure [33–35]. Second, this study was in healthy volunteers with all but 1 participant having VExUS zero at baseline limiting our ability to apply these results to patients with hemodynamic derangement. Because the normal heart seeks to keep the right atrial pressure low, our observations are somewhat expected. This is in distinction to Hermansen and colleagues who performed VExUS in ventilated, sedated, post-cardiac surgery patients during the semi-recumbent and legs raised positions [28]. Contrary to our findings, they did observe a clinically significant change in renal and hepatic Doppler morphology consistent with increasing venous congestion. Nevertheless, given that the jugular vein Doppler morphology revealed robust changes in our healthy volunteer study, we suspect that jugular Doppler would also disclose preload changes in the post-surgical group. Third, interpretation of the jugular Doppler waveform does have a subjective element. We tried to minimize this effect by having three experts in venous Doppler interpretation blindly and independently score the signals. Of 41 files, there were only 5 instances where the 3 reviewers were not unanimous in their blind and independent interpretation. All five of these instances had agreement between two reviewers and the third reviewer judged the jugular morphology differently by only a single ordinal value (i.e., 0 versus 1 or 1 versus 2). This was also reflected in the Fleiss Kappa coefficient (i.e., 0.83) which indicated high inter-rater agreement between the three blinded experts. Finally, we did not measure stroke volume (SV) during preload changes in this healthy cohort. Previous work using venous measures (e.g., IVC collapse and central venous pressure) to infer ‘preload responsiveness’ show false positive rates of 20–30% [36–38]. In other words, a collapsing IVC or low right atrial pressure is observed in 1-to-2 out of 5 patients who are, nevertheless, fluid unresponsive; such physiology is anticipated in patients with a shallow cardiac function curve [6, 39]. Thus, we are skeptical that pure venous measures will exactly predict SV response. Furthermore, some investigators have observed high preload unresponsive rates in healthy volunteers [40], which is contrary to our earlier work. In over 30 healthy volunteers and 70 preload augmentations, 100% of healthy subjects increased SV by at least 10% [18, 21]. In our previous studies, the change in SV was monitored over thousands of cardiac cycles which may be a limitation of other investigations [41]. With respect to the current study, relating change in carotid arterial Doppler (i.e., as a surrogate for changing SV [42]) to venous measures in healthy subjects during gravity-induced preload change is an active avenue of investigation.

## Conclusions

In healthy volunteers, jugular Doppler waveforms measured using a wearable Doppler device had high diagnostic accuracy for gravity-induced preload changes. In addition, the supine position showed the highest agreement between the jugular and hepatic Doppler waveforms. Finally, patient position had little effect on the overall VExUS score. The clinical implications of these findings are that internal jugular vein Doppler morphology may serve as a rapid method to phenotype patients in shock, and determine the physiological effect of preload during functional hemodynamic monitoring. Furthermore, patient position in the hospital bed does not significantly affect VExUS score, at least in relatively healthy, euvolemic, subjects with intact cardiovascular reflexes. Further investigation in patients with hemodynamic pathology is needed to confirm these preliminary observations.

## Data Availability

The data sets used and/or analysed during the current study are available from the corresponding author on reasonable request.

## Abbreviations

IV: Intravenous
VExUS: Venous excess ultrasound score
IVC: Inferior vena cava
S-wave: Systolic wave
D-wave: Diastolic wave
B-mode: Brightness mode
JVP: Jugular venous pressure
